# The Impact of Neutrophil-to-Lymphocyte Ratio after Two Courses of Pembrolizumab for Oncological Outcomes in Patients with Metastatic Urothelial Carcinoma

**DOI:** 10.3390/biomedicines10071609

**Published:** 2022-07-06

**Authors:** Risa Tomioka-Inagawa, Keita Nakane, Torai Enomoto, Masayuki Tomioka, Tomoki Taniguchi, Takashi Ishida, Kaori Ozawa, Kimiaki Takagi, Hiroki Ito, Shinichi Takeuchi, Makoto Kawase, Kota Kawase, Daiki Kato, Manabu Takai, Koji Iinuma, Shigeaki Yokoi, Masahiro Nakano, Takuya Koie

**Affiliations:** 1 Department of Urology, Gifu University Graduate School of Medicine, Gifu 5011194, Japan; risa_tom@gifu-u.ac.jp (R.T.-I.); keitaco@gifu-u.ac.jp (K.N.); gallxy7@gifu-u.ac.jp (S.T.); buki2121@gifu-u.ac.jp (M.K.); stnf55@gifu-u.ac.jp (K.K.); andreas7@gifu-u.ac.jp (D.K.); takai_mb@gifu-u.ac.jp (M.T.); kiinuma@gifu-u.ac.jp (K.I.); 2 Department of Urology, Matsunami General Hospital, Hashima-gun 5016062, Japan; try@mghg.jp; 3 Department of Urology, Japanese Red Cross Takayama Hospital, Takayama 5068550, Japan; tomiokam@gifu-u.ac.jp; 4 Department of Urology, Chuno Kosei Hospital, Seki 5013802, Japan; tomokidbx@gmail.com; 5 Department of Urology, Ogaki Municipal Hospital, Ogaki 5038502, Japan; k.ohzawa.lily@gmail.com; 6 Department of Urology, Gifu Municipal Hospital, Gifu 5008513, Japan; justaskaxis@gmail.com; 7 Department of Urology, Daiyukai Daiichi Hospital, Ichinomiya 4918551, Japan; kimiaki_takagi5619@yahoo.co.jp; 8 Department of Urology, Toyota Memorial Hospital, Toyota 4718513, Japan; seanoel2@gmail.com; 9 Department of Urology, Central Japan International Medical Center, Minokamo 5058510, Japan; s-yokoi@cjimc-hp.jp; 10 Department of Urology, Gifu Prefectural General Medical Center, Gifu 5008717, Japan; mnakano@gifu-hp.jp

**Keywords:** multicenter cohort study, oncological outcomes, pembrolizumab, metastatic urothelial carcinoma, neutrophil-to-lymphocyte ratio

## Abstract

We focused on the therapeutic effect of pembrolizumab for metastatic urothelial carcinoma (mUC) and evaluated predictive factors for improving clinical outcomes. We conducted a retrospective multicenter cohort study of patients with mUC who received pembrolizumab. The endpoint was to evaluate the association between clinicopathological features and oncological outcomes. A total of 160 patients were enrolled in this study and were divided into two groups: the responder and the non-responder group, according to the best response. They were followed up for a median period of 10 months. The median overall (OS) and progression-free survival (PFS) in this study were 17 and 4 months, respectively. The responder group did not achieve median OS and it was 10 months in the non-responder group (*p* < 0.001). Similarly, the responder group did not achieve PFS, and it was 2 months in the non-responder group (*p* < 0.001). Regarding the neutrophil-to-lymphocyte ratio (NLR) after two courses of administration of pembrolizumab, patients with NLR < 3.24 had significantly better oncological outcomes than those with NLR ≥ 3.24. Multivariate analysis showed a significant association between NLR after two courses of pembrolizumab and OS. Therefore, the absolute value of NLR after two courses of pembrolizumab was a significant predictive factor for oncological outcomes.

## 1. Introduction

Metastatic urothelial carcinoma (mUC) is an aggressive urological cancer, and the prognosis of patients with mUC remains poor, with a 5-year overall survival (OS) of only 4.6% [1,2]. Although platinum-based combination chemotherapy has been the first-line systemic treatment for mUC over the last two decades, the median OS has been reported to be 12–15 months [1,3,4]. In addition, approximately half of the patients with mUC may be ineligible for cisplatin-based chemotherapy; therefore, these patients receive carboplatin-based regimens, which means that may have further potential disadvantages in oncological outcomes [5].

Pembrolizumab is a highly selective, humanized monoclonal IgG4ĸ isotype antibody against programmed death 1 (PD-1) that showed antitumor activity in patients with advanced urothelial carcinoma (UC) in the KEYNOTE-012 and KEYNOTE-052 clinical trials [6,7]. In the randomized, open-label, phase III KEYNOTE-045 trial, pembrolizumab as second-line therapy had better oncological outcomes and objective response rate (ORR) than antitumor agents in patients with advanced UC who progressed during or after platinum-based chemotherapy [8]. In addition, the rate of treatment-related adverse events (AEs) of any grade was lower with pembrolizumab than with other anticancer agents [8]. In the KEYNOTE-045 trial, with a follow-up period of >2 years, patients with advanced UC treated with pembrolizumab had higher median OS rates at 1 and 2 years, ORR, and median duration of response (DOR), compared with those treated with chemotherapy [9]. Currently, pembrolizumab is recommended as the second-line treatment for mUC according to several guidelines [3,10,11].

In recent studies, neutrophil-to-lymphocyte ratio (NLR) [12], psoas muscle mass index (PMI) [13], albumin-to-globulin ratio (AGR) [14], C-reactive protein (CRP) flare response [15], poor Eastern Cooperative Oncology Group performance status (ECOG-PS) [16], number of metastatic sites, and an early increase in NLR [17] have been useful predictive markers for oncological outcomes in patients with mUC treated with pembrolizumab [12,13,14,15,16,17]. Fukuokaya et al. reported that continuous administration of pembrolizumab until disease progression may improve OS in patients with mUC [18]. However, the number of patients with mUC enrolled in these studies was relatively small. To date, the optimal treatment strategy with pembrolizumab for patients with mUC in regard to who should continue or stop pembrolizumab treatment remains unclear. Regarding patients with mUC who received pembrolizumab as second-line therapy, we focused on the therapeutic effect of pembrolizumab for mUC and evaluated the predictive factors to improve clinical outcomes.

## 2. Materials and Methods

### 2.1. Patients

This study was conducted with the approval of the Institutional Review Board of Gi-fu University (authorization number: 2021-B080). The requirement for patient consent was waived because of the retrospective study design. According to the provisions of the ethics committee and ethics guidelines in Japan, the study information is disclosed to the public in the case of retrospective and/or observational studies, with materials, such as existing documentation. The details of this study are available at http://www.med.gifu-u.ac.jp/file/2020-271.pdf. (Accessed on 13 May 2022)

We conducted a retrospective, multicenter cohort study of patients with mUC who received pembrolizumab at 10 institutions in Japan between December 2017 and August 2021. All of the enrolled patients had histologically confirmed UC with distant metastases and had received platinum-based chemotherapy before pembrolizumab administration. Clinicopathological and laboratory parameters included patient age, sex, height, weight, ECOG-PS, smoking history, primary tumor site, metastatic sites, patients who underwent definitive therapy for the primary site, hemoglobin level (Hb), serum albumin level (Alb), CRP, and NLR. In all cases, tumor staging was based on the American Joint Committee on Cancer’s 8th Edition Cancer Staging Manual [19].

### 2.2. Treatment Schedule

All participants had previously received platinum-based chemotherapy and then subsequently their UC progressed. Pembrolizumab was administered as a 3-weekly (200 mg) regimen, based on the schedule reported in a clinical trial [7]. Pembrolizumab was continued until radiographic progression, refusal of treatment by the patient, or intolerance to treatment-related AEs according to the National Cancer Institute Common Terminology Criteria for Adverse Events (version 5.0) [20].

### 2.3. Patient Evaluation

Baseline data comprised complete history taking, physical examination, and chest, abdominal, and pelvic computed tomography (CT). In all patients, CT was performed at a 2-month interval, till disease progression was radiologically proven. The best overall response (BOR) was evaluated based on the Response Evaluation Criteria in Solid Tumors (RECIST) guidelines, version 1.1 [21], as complete response (CR), partial response (PR), stable disease (SD), or progressive disease (PD). NLR was calculated by dividing the absolute neutrophil count by the absolute lymphocyte count in the peripheral blood. The cutoff values for Hb, Alb, CRP, and NLR were derived from the area under the receiver operating characteristic (ROC) curve [22] and defined as the minimal values for (1 − sensitivity)^2^ + (1 − specificity)^2^. In addition, the patients were divided into two groups according to the BOR: those who achieved CR or PR (responder group) or SD or PD (non-responder group).

### 2.4. Endpoints and Statistical Analysis

The primary endpoint was the association between clinicopathological features and oncological outcomes. Oncological outcomes, including OS and progression-free survival (PFS), were defined as the secondary endpoints. The software JMP 14 (SAS Institute Inc., Cary, NC, USA) was used for data analysis. Continuous and categorical variables were compared using the Kruskal–Wallis test. The first date of pembrolizumab treatment was the starting point to estimate OS and PFS. OS and PFS were defined as the time from pembrolizumab treatment initiation to all-cause death and disease progression, respectively. Disease progression was defined as the appearance of locoregional disease or distant metastasis. These were evaluated using the Kaplan-Meier method, and differences were assessed according to clinical variables using the log-rank test. A Cox proportional hazards model was used for multivariate analysis. All two-sided *p* values of <0.05 were considered statistically significant.

## 3. Results

### 3.1. Patient Characteristics

In total, 211 patients were enrolled in this study. Overall, 26 patients were excluded from the study due to missing data (11 patients) and lack of assessment of the therapeutic effect after pembrolizumab administration (14 patients). This meant that 160 patients were enrolled and analyzed in this study.

Table 1 summarizes the demographic data for patients of two groups classified according to the BOR. The median age of the patients was 72 years (interquartile range [IQR], 69–78 years). The median body mass index was 22.2 (IQR, 19.6–24.5). In this cohort, 75.4% of the patients were male, 61.3% had a smoking history, and 13.4% of the patients had ECOG-PS ≥ 2. The most common primary lesions and metastatic sites were the urinary bladder (54.9%) and the lymph nodes (73.2%). A total of 121 patients (85.9%) underwent definitive therapy for the primary lesions.

The blood biochemical findings before and after two courses of pembrolizumab are shown in Table 2. Although CRP, Alb, Hb, and NLR levels were within normal limits in all patients before and after pembrolizumab administration, pretreatment CRP and Hb levels in the non-responder group were significantly different from those in the responder group. Conversely, CRP, Alb, Hb, and NLR after two courses of pembrolizumab in the responder group were significantly different from those in the non-responder group.

### 3.2. Oncological Outcomes

**Figure 1:**
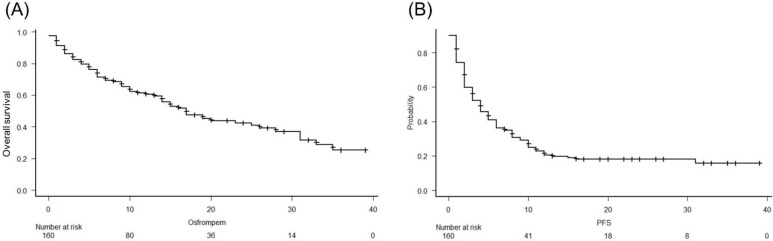
Analysis of overall survival (OS) (**A**) and progression-free survival (PFS) (**B**) using the Kaplan-Meier method in patients with metastatic urothelial carcinoma treated with pembrolizumab. The median OS and PFS in this study were 17 months and 4 months, respectively.

The median follow-up period was 10 months (IQR, 5–19 months). At the end of the follow-up period, 81 patients (50.6%) died from UC. The median OS and PFS in this study were 17 months (95% confidence interval [CI], 15–26 months) and 5 months (95% CI, 3–5 months), respectively (Figure 1A,B).

The BORs in changes in CRP, albumin, hemoglobin, and NLR levels, before and after pembrolizumab administration, are listed in Table 3. Before pembrolizumab administration, CRP levels were significantly lower in the responder group compared to the non-responder group (*p* = 0.001), even though albumin, hemoglobin, and NLR levels exhibited no significant differences between the two groups (Table 3). Conversely, all covariates after pembrolizumab administration were significantly improved in the responder group compared to the non-responder group (Table 3).

**Figure 2:**
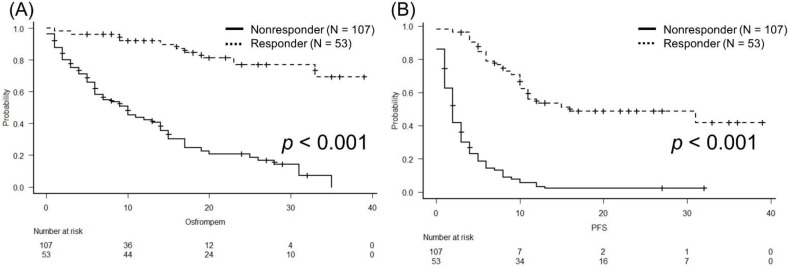
Analysis of overall survival (OS) (**A**) and progression-free survival (PFS) (**B**) according to best overall response, using the Kaplan-Meier method, in patients with metastatic urothelial carci-noma treated with pembrolizumab. The responder group did not achieve median OS and the non-responder group had a median OS of 10 months (*p* < 0.001). The median PFS in the responder group was not reached and it was reached at two months in the non-responder group (*p* < 0.001).

According to the BOR, one- and two-year OS values were 69.1% and 53.6%, respectively, in the responder group. In the non-responder group, these values were 47.0% and 24.9%, respectively (Figure 2A; *p* < 0.001). The responder group did not achieve PFS, and it was reached at two months in the non-responder group (Figure 2B; *p* < 0.001).

**Figure 3:**
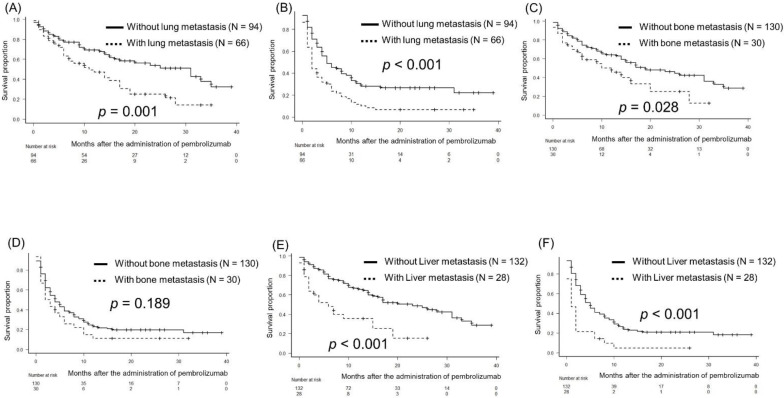
Kaplan-Meier curves illustrating overall (OS) and progression-free survival (PFS): (**A**,**B**) for with/without lung metastasis, (**C**,**D**) for with/without bone metastasis, and (**E**,**F**) for with/without liver metastasis.

Regarding lung metastasis, the median OS and PFS were 31 months and 5 months in patients without lung metastasis and 11 months and 2 months in those with lung metastasis (*p* = 0.001 and *p* < 0.001, respectively; Figure 3A and 3B, respectively). Regarding bone metastasis, the median OS was 19 months in patients without bone metastasis and 12 months in those with bone metastasis (*p* = 0.028; Figure 3C). The median PFS was 4 months in patients without bone metastasis and 2.5 months in those with bone metastasis (*p* = 0.189; Figure 3D). Regarding liver metastasis, the median OS and PFS were 23 months and 5 months in patients without liver metastasis and 6 months and 2 months in those with liver metastasis (*p* < 0.001 and *p* < 0.001, respectively; Figure 3E,F), respectively.

**Figure 4:**
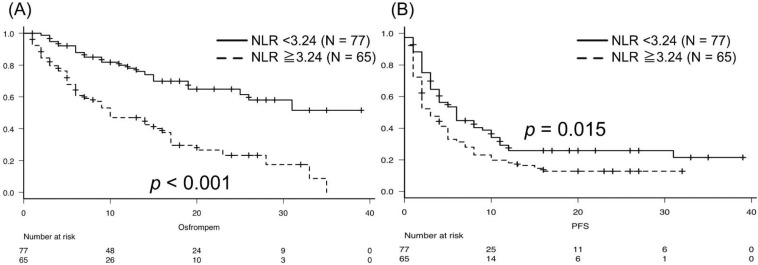
According to NLR, patients with NLR < 3.24 did not achieve the median OS, and those with NLR ≥ 3.24 had a median OS of 10 months (*p* < 0.001; (**A**)). The median PFS was 6 months in patients with NLR < 3.24 and 3 months in patients with NLR ≥ 3.24 (*p* = 0.015; (**B**)).

Patients with NLR < 3.24 after two courses of pembrolizumab did not achieve the median OS, and those with NLR ≥ 3.24 had a median OS of 10 months (*p* < 0.001; Figure 4A). The median PFS was 6 months in patients with NLR < 3.24 and 3 months in patients with NLR ≥ 3.24 (*p* = 0.015; Figure 4B).

In the multivariate analysis, it was shown that NLR and CRP after two courses of pembrolizumab and lung and liver metastases were significantly associated with OS (Table 4).

According to the association between NLR and statistically correlated variables, CRP, albumin, and hemoglobin levels were significantly associated with NLR. However, there were no significant differences between NLR and ECOG-PS, and liver and bone metastases.

## 4. Discussion

Based on the KEYNOTE-045 trial, several guidelines recommended pembrolizumab as second-line therapy for mUC [3,10,11]. In addition, the ORR and DOR in patients with mUC who received pembrolizumab were superior to those in patients treated with chemotherapy [9]. Recently, Bellmunt et al. reported the 5-year follow-up data from the KEYNOTE-045 trial at the 2021 ASCO annual meeting [23]. The median OS was longer for pembrolizumab than for chemotherapy (10.1 vs. 7.2 months; hazard ratio [HR], 0.71 [95% CI, 0.59–0.86]) [23]. In addition, the median DOR for responders was significantly longer for pembrolizumab (29.7 months) than for chemotherapy (4.4 months) [23]. This study concluded that pembrolizumab maintained clinically meaningful OS benefits in patients with locally advanced or mUC who progressed during or after platinum-based chemotherapy [23]. Fukuokaya et al. reported that the median OS was significantly longer in the continuation group than in the discontinuation of pembrolizumab group (17.8 vs. 8.8 months; *p* = 0.038) [18]. In the multivariate Cox regression model, continued pembrolizumab administration and longer duration of pembrolizumab treatment beyond progression were independently associated with a reduced risk of all-cause mortality [18]. These results suggest that the oncological outcomes of patients with mUC who received pembrolizumab as second-line treatment are not necessarily acceptable. In addition, unnecessarily long-term continued pembrolizumab administration may lead to disease progression or loss to third-line therapy in patients with mUC. Therefore, predictive biomarkers need to be established to determine whether patients with mUC should continue to receive pembrolizumab therapy.

Furthermore, it is very important to identify predictive markers for achieving maximal benefit with prolonged oncological outcomes and therapeutic responses in patients with mUC who have received immune checkpoint inhibitors, including pembrolizumab. For patients with mUC treated with pembrolizumab, several predictive biomarkers have been identified. Shimizu et al. reported that sarcopenia was an independent predictor of OS in multivariate analysis [13]. Furthermore, a decrease in PMI ≥ 5% in a month timescale was an independent predictor of PFS and OS in patients with mUC who received pembrolizumab [13]. Regarding AGR, Kaplan-Meier curves with log-rank tests showed a significant association of AGR < 0.95 and NLR ≥ 3 with shorter PFS and OS [14]. Multivariate Cox proportional hazard regression analysis identified pretreatment AGR < 0.95 as an independent predictor of poor prognosis for PFS [14]. Ogihara et al. reported that the pretreatment NLR was an independent indicator of disease progression and cancer-specific death [12]. In a multicenter retrospective analysis, the CAN score, which is based on the number of items using CRP, Alb, and NLR, was a useful predictive marker for shorter OS [24]. However, these studies identified predictive factors for the effectiveness of pembrolizumab using pretreatment parameters. Therefore, the discontinuation criteria for pembrolizumab in mUC patients remains unclear.

In contrast, the decreased NLR after pembrolizumab treatment may be a useful biomarker for other carcinomas [25]. Regarding non-small cell lung cancer, high post-treatment NLR, liver metastasis, and brain metastasis are independent prognostic factors for shorter PFS [26]. A high post-treatment NLR was identified as an independent prognostic factor for OS in a multivariate analysis [26]. They concluded that NLR at six weeks after treatment initiation was a prognostic marker in patients with advanced lung cancer treated with anti-PD-1 antibody [26]. Lalani et al. investigated the utility of NLR in patients with metastatic renal cell carcinoma treated with immune checkpoint inhibitors [27]. A relative percent change of ≥25% in NLR from baseline to 6 weeks after treatment reduced ORR, revealing it to be an independent prognostic factor for PFS and OS [27]. For patients with mUC, a ≥25% decrease in NLR level from the baseline to post-treatment was significantly associated with lower disease progression and cancer-specific death rates, unlike their counterparts [12]. Tamura et al. reported that poor ECOG-PS, two or more metastatic organs, and higher relative NLR change 6 weeks after the initiation of pembrolizumab were identified as independent predictors of OS in the multivariate analysis [16]. To the best of our knowledge, this study is the first to report that the absolute value of NLR after two courses of pembrolizumab immune therapy was significantly correlated with oncological outcomes in patients with mUC. Patients with mUC who showed an improved NLR after the administration of pembrolizumab could have a possible beneficial effect with ≥3 courses of pembrolizumab therapy because of the enhancement of the immune response.

First, the study design was retrospective, using multicenter data. Therefore, the enrollment of the study population might have resulted in selection bias, and diagnostic and therapeutic data might have varied among these institutions. Second, the relatively small sample size and short follow-up period might also influence the strength of our findings. Third, the study lacked a control group of patients receiving chemotherapy with anticancer agents for mUC. Finally, we did not collect data on the immune-related AEs following pembrolizumab treatment.

## 5. Conclusions

In this study, NLR was a significantly useful predictive factor for oncological outcomes. Further prospective studies and long-term evaluations in large patient populations are required to identify useful predictive markers for determining patients with mUC who should continue to receive pembrolizumab for a relatively long term.

## Figures and Tables

**Table 1:** Patient characteristics.

Variables	Responder	Non-Responder	*p*
Number	53	107	
Age (year, median, IQR)	72 (70–78)	72 (68–78)	0.707
Sex (n, %)			0.845
Male	41 (77.4)	80 (74.8)	
Female	12 (22.6)	27 (25.2)	
BMI (kg/m^2^, median, IQR)	22.2 (19.4–24.2)	22.0 (19.5–24.5)	0.988
ECOG-PS			
0	38 (71.7)	56 (52.3)	0.026
1	12 (22.6)	29 (27.1)	
2	1 (1.9)	16 (15.0)	
3	2 (3.8)	6 (5.6)	
Primary site (number, %)			0.485
Upper urinary tract	19 (35.8)	30 (28.0)	
Bladder	26 (49.1)	63 (58.9)	
Upper urinary tract and Bladder	8 (15.1)	14 (13.1)	
Location of metastases (number, %)			
Lung	13 (24.5)	53 (49.5)	0.004
Liver	5 (9.4)	23 (21.5)	0.077
Bone	5 (9.4)	25 (23.4)	0.051
Lymph node	36 (67.9)	80 (74.8)	0.452
Smoking history (number, %)			
Never	18 (34.0)	43 (40.2)	0.239
Current	5 (9.4)	18 (16.8)	
Former	30 (56.6)	46 (43.0)	
Definitive therapy for primary site (number, %)	47 (88.7)	83 (77.6)	0.131
Histological subtype (number, %)			0.322
Pure urothelial carcinoma	43 (82.7)	95 (88.8)	
Sarcomatoid variant	3 (5.7)	3 (2.8)	
other variants	7 (13.2)	9 (17.3)	
Follow-up period	19 (12–26)	6 (3–13)	<0.001
(months, median, IQR)			

IQR, interquartile range; ECOG-PS, Eastern Cooperative Oncology Group performance status.

**Table 2:** Clinical covariates before and after two courses of pembrolizumab.

Variables	Responder	Non-Responder	*p*
Number	53	107	
Before the administration of pembrolizumab			
CRP (mg/dL, median, IQR)	0.20 (0.10–1.40)	1.14 (0.23–2.77)	0.002
Albumin (g/dL, median, IQR)	3.90 (3.50–4.12)	3.60 (3.20–3.95)	0.006
Hemoglobin (g/dL, IQR)	11.10 (9.20- 12.60)	10.50 (9.12–12.00)	0.133
NLR (median, IQR)	2.94 (2.07–4.47)	2.96 (2.08–4.96)	0.843
After two courses of pembrolizumab			
CRP (mg/dL, IQR)	0.14 (0.07–0.46)	1.05 (0.42–3.83)	<0.001
Albumin (g/dL, IQR)	3.90 (3.68–4.23)	3.60 (3.23–3.98)	<0.001
Hemoglobin (g/dL, IQR)	11.90 (10.88- 13.40)	10.80 (9.50–12.30)	0.003
NLR (median, IQR)	2.13 (1.60–3.50)	3.30 (2.50–5.80)	<0.001

CRP, C-reactive protein; IQR, interquartile range; NLR, neutrophil-to-lymphocyte ratio.

**Table 3:** Changes in CRP, Albumin, Hemoglobin, and NLR levels, before and after pembrolizumab administration.

	Best of response					
Variables		CR	PR	SD	PD	*p*
Number		23	30	33	74	
**Before pembrolizumab administration**						
CRP, <0.63 mg/dL		19 (82.6)	18 (60.0)	17 (51.5)	26 (35.1)	*0.001*
CRP, ≥0.63 mg/dL		4 (17.4)	12 (40.0)	16 (48.5)	48 (64.9)	
Albumin, <3.7 g/dL		6 (26.1)	11 (37.9)	16 (48.5)	44 (59.5)	*0.024*
Albumin, ≥3.7 g/dL		17 (73.9)	18 (62.1)	17 (51.5)	30 (40.5)	
Hemoglobin, <10.6 g/dL		6 (26.1)	14 (46.7)	16 (48.5)	40 (54.8)	*0.122*
Hemoglobin, ≥10.6 g/dL		17 (73.9)	16 (53.3)	17 (51.5)	33 (45.2)	
NLR, <4.89		19 (82.6)	21 (70.0)	28 (84.8)	51 (68.9)	*0.243*
NLR, ≥4.89		4 (17.4)	9 (30.0)	5 (15.2)	23 (31.1)	
**After pembrolizumab administration**						
CRP, <0.67 mg/dL		20 (90.9)	23 (79.3)	18 (58.1)	15 (25.0)	*<0.001*
CRP, ≥0.67 mg/dL		2 (9.1)	6 (20.7)	13 (41.9)	45 (75.0)	
Albumin, <3.7 g/dL		5 (21.7)	8 (27.6)	13 (40.6)	40 (64.5)	*<0.001*
Albumin, ≥3.7 g/dL		18 (78.3)	21 (72.4)	19 (59.4)	22 (35.5)	
Hemoglobin, <10.0 g/dL		0 (0)	6 (20.7)	12 (37.5)	22 (36.1)	*0.004*
Hemoglobin, ≥10.0 g/dL		23 (100.0)	23 (79.3)	20 (62.5)	39 (63.9)	
NLR, <3.24		18 (81.8)	19 (65.5)	16 (51.6)	24 (40.0)	*0.004*
NLR, ≥3.24		4 (18.2)	10 (34.5)	15 (48.4)	36 (60.0)	

CR, complete response; PR, partial response; SD, stable disease; PD, progressive disease; CRP, C-reactive protein; NLR, neutrophil-to-lymphocyte ratio.

**Table 4:** Univariate and multivariate Cox proportional hazard regression analysis according to overall survival.

Variables	Univariate			Multivariate		
	HR	95% CI	*p*	HR	95% CI	*p*
Age, ≥75 vs. <75	0.91	0.57–1.44	0.705			
Sex, Female vs. Male	0.87	0.51–1.48	0.610			
ECOG-PS, ≥2 vs. ≤1	1.37	1.07–1.75	0.013	1.88	0.95–3.72	0.068
BMI, <21.9 vs. ≥21.9	0.82	0.53–1.27	0.378			
Smoking history, yes vs. no	0.74	0.47–1.16	0.201			
Lung metastasis, yes vs. no	1.98	1.28–3.07	0.002	2.02	1.15–3.55	0.014
Liver metastasis, yes vs. no	2.56	1.55–4.24	<0.001	2.65	1.35–5.17	0.004
Bone metastasis, yes vs. no	1.82	1.1–3.03	0.021	1.11	0.61–2.00	0.720
Lymph node metastasis, yes vs. no	0.95	0.6–1.51	0.830			
Surgery of the primary site, yes vs. no	0.63	0.37–1.07	0.084			
Radiation therapy of the primary site, yes vs. no	1.17	0.43–3.21	0.760			
Albumin, ≥3.7 vs. <3.7	0.28	0.23–0.56	<0.001	0.59	0.30–1.15	0.126
CRP, ≥0.67 vs. <0.67	1.08	1.04–1.11	<0.001	2.28	1.13–4.57	0.020
Hemoglobin, ≥10.0 vs. <10.0	0.29	0.18–0.48	<0.001	1.08	0.58–2.02	0.790
NLR, ≥3.24 vs. <3.24	3.21	1.94–5.33	<0.001	2.82	1.50–5.31	0.001

HR, hazard ratio; CI, confidence interval; ECOG-PS, Eastern Cooperative Oncology Group Performance Status; BMI, body mass index; CRP, C-reactive protein; NLR, neutrophil-to-lymphocyte ratio.

## Data Availability

Requests for the data presented in this study should be addressed to the corresponding author. The data are not publicly available for privacy and ethical reasons.
